# Acid catalyzed cyclodimerization of 2,2-bis(trifluoromethyl)-4-alkoxy-oxetanes and -thietanes. Synthesis of 2,2,6,6-tetrakis(trifluoromethyl)-4,8-dialkoxy-1,5-dioxocanes and 3,3,7,7-tetrakis(trifluoromethyl)-9-oxa-2,6-dithia-bicyclo[3.3.1]nonane

**DOI:** 10.3762/bjoc.6.46

**Published:** 2010-05-10

**Authors:** Viacheslav A Petrov, Will Marshall

**Affiliations:** 1 DuPont Central Research and Development (publication No 8966), Experimental Station, PO Box 800500, Wilmington DE 19880-0500, United States; 2 DuPont Corporate Center for Analytical Sciences, Experimental Station, PO Box 800500, Wilmington DE 19880-0500, United States

**Keywords:** cyclodimerization, electrophilic [4 + 4] cyclodimerization, fluorinated oxetanes, fluorinated thietanes, reaction with alcohols, reaction with H_2_SO_4_

## Abstract

Treatment of 2,2-bis(trifluoromethyl)-4-R-oxetanes (R = C_2_H_5_O, *n*-C_3_H_7_O, *n*-C_4_H_9_O) with BF_3_·OEt_2_ in CH_2_Cl_2_ solvent results in spontaneous electrophilic [4 + 4] cyclodimerization with the formation of the corresponding 2,2,6,6-tetrakis(trifluoromethyl)-4,8-dialkoxy-1,5-dioxocanes, isolated in 31–42% yield. The structures of two products (R = C_2_H_5_O and *n*-C_3_H_7_O) were established by single crystal X-ray diffraction. The corresponding oxetane carrying the bulky *t*-C_4_H_9_O group has different reactivity towards BF_3_·OEt_2_, slowly producing a mixture of two acyclic, unsaturated products.

Clean and spontaneous reaction with alcohols is another interesting transformation of oxetanes described in this paper. The reaction leads to high yield formation of the corresponding acetals (CF_3_)_2_C(OH)CH_2_CH(OR)OR′.

Structurally related 2,2-bis(trifluoromethyl)-4-R-thietanes (R = *i*-C_3_H_7_O, *t*-C_4_H_9_O and C_2_H_5_O) have different reactivity towards electrophiles. They are totally inert to the action of BF_3_·OEt_2_ and rapidly react with a protic acid (H_2_SO_4_) forming the same product, 3,3,7,7-tetrakis(trifluoromethyl)-9-oxa-2,6-dithia-bicyclo[3.3.1]nonane in 35–50% yield. The structure of this product was established by single crystal X-ray diffraction.

## Introduction

Polyfluorinated 2,2-bis(trifluoromethyl)-4-alkoxy-oxetanes and -thietanes are readily available materials, prepared by [2 + 2] cycloaddition of vinyl ethers with hexafluoroacetone [1–3] or hexafluorothioacetone [4–5], respectively. Although both groups of compounds have been known for over 40 years, reports on their chemical transformations are limited. Among the reported reactions of oxetanes are hydrolysis of 2,2-bis(trifluoromethyl)-4-alkoxyoxetanes **1** leading to the formation of 4,4,4-trifluoro-3-(trifluoromethyl)-3-hydroxybutanal [1,6] and thermal or acid catalyzed isomerization of 2,2-bis(trifluoromethyl)-4-*n*-butoxyoxetane into (*E*)-4-*n*-butoxy-1,1,1-trifluoro-2-(trifluoromethyl)but-3-en-2-ol [2].

Some compounds containing two 2,2-bis(trifluoromethyl)oxetane units, such as bis-4,4-(trifluoromethyl)oxetan-2-yl ether, were reported to undergo Lewis acid catalyzed polymerization [3].

Known reactions of 2,2-bis(trifluoromethyl)-4-alkoxythietanes (R = CH_3_O and C_2_H_5_O) include the formation of 4-(4,4-bis(trifluoromethyl)thietan-2-yloxy)-2,2-bis(trifluoromethyl)thietane on treatment with H_2_SO_4_ [4], thiophilic ring opening by the action of alkyl magnesium or lithium reagents [4], the recently reported oxidation with selective formation of the corresponding S-oxides [7], and an unusual reductive ring expansion leading to the corresponding dihydrothiophenes [7].

As part of a program to identify new, readily available fluorinated monomers, we have carried out a comparative study of the reactivity of 2,2-bis(trifluoromethyl)-4-alkoxy- oxetanes and -thietanes towards acids. The results of this study are reported in this paper.

## Results and Discussion

In sharp contrast to the reported isomerization of 2,2-bis(trifluoromethyl)-4-alkoxyoxetanes catalyzed by protic acids [2], the reaction of oxetanes **1a–c** with a catalytic amount of *Lewis acid* leads to a completely different reaction course. The addition of boron trifluoride etherate catalyst to a solution of the oxetane in dichloromethane resulted in a spontaneous and mildly exothermic reaction. A very interesting feature of this process is the appearance of highly intensive blue or blue-green colour upon the addition of the first drop of the catalyst. The colour of the reaction mixture rapidly changes to dark red and finally to brown and, at this stage, usually the formation of a precipitate is observed. The solid products **2a–c** were isolated in moderate yields after filtration of the cold reaction mixture and washing of the filter cake with water. Analytically pure samples were prepared by crystallization from hexane. ^1^H, and ^13^C NMR and IR spectroscopic data show the absence of a C=C bond in all of the isolated products. Due to the fact, that ^1^H and ^19^F NMR spectra of **2a**, **b** (see Supporting Information File 1, Table 1, Entries 1, 2) and starting oxetanes **1a**, **b** have a similar appearance, spectroscopic data were not sufficient for an unambiguous assignment of the structure. Consequently, structure assignments for **2a** and **2b** were made based on single crystal X-ray diffraction data (see Supporting Information File 2).

**Scheme 1:**
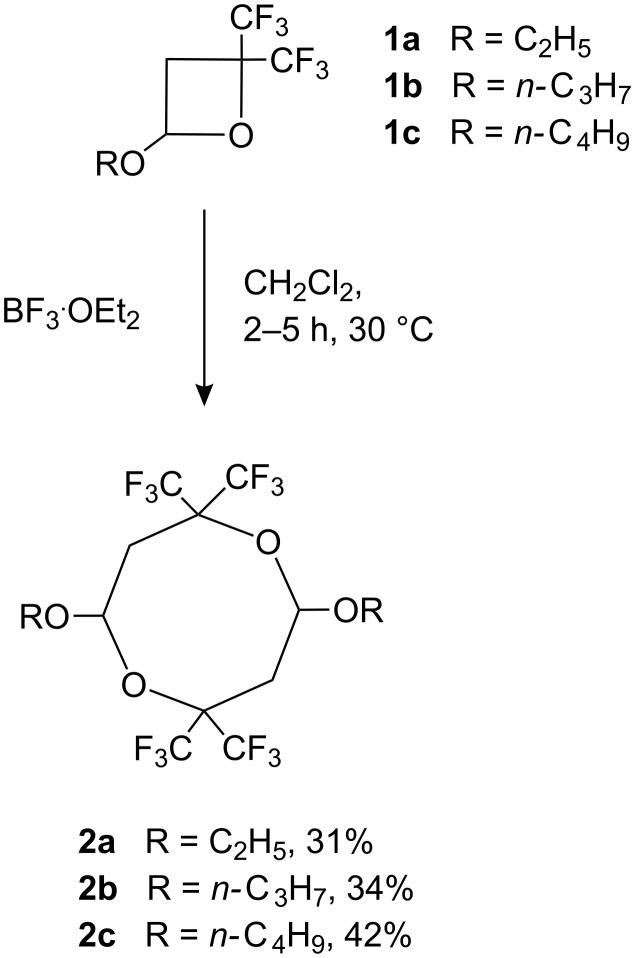
Electrophilic [4 + 4] dimerization of oxetanes **1a–c** under action of BF_3_·OEt_2_ catalyst.

**Figure 1:**
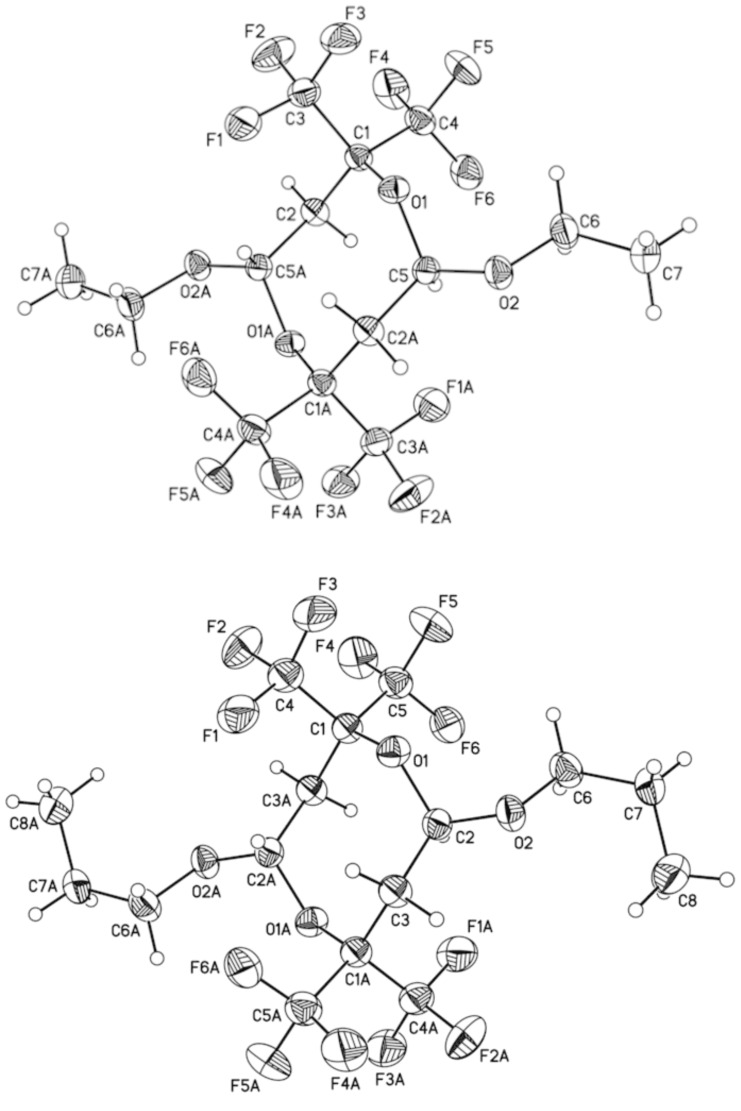
ORTEP drawing of compounds **2a** and **2b** with thermal ellipsoids drawn to the 50% probability level.

Both compounds were found to have a symmetrical 2,2,6,6-tetrakis(trifluoromethyl)-1,5-dioxocane core with a trans-orientation of two alkoxy groups located at positions 4 and 8 (Scheme 1 and Figure 1 for structure of **2a** and **2b**, Supporting Information File 2 for single crystal X-ray data).

Since ^1^H, ^13^C and ^19^F NMR spectra of compound **2c** were similar to the NMR spectra of **2a**, **b**, it is assumed that compound **2c** also has a 2,2,6,6-tetrakis(trifluoromethyl)-1,5-dioxocane structure.

Despite the fact that yields of 1,5-dioxocanes **2a–c** in the reaction of oxetanes with BF_3_·OEt_2_ are modest, the process itself is simple, reproducible and provides easy access to this new group of stable polyfluorinated 1,5-dioxocanes. It should also be pointed out, that examples of electrophilic [4 + 4] cycloaddition reactions are extremely rare and limited to two examples: the reaction of the oxetane (derived from the cycloaddition of 1,1-dimethoxyethylene and 2,2-dimethylcyclopropanone), leading to a stable hydrocarbon 1,5-dioxocane [8] and the formation of the corresponding fluorinated 1,5-dioxocane intermediate [9–10] observed in the isomerization of 2-ethoxy-4-(perfluoropropan-2-ylidene)oxetane [11–12].

**Scheme 2:**
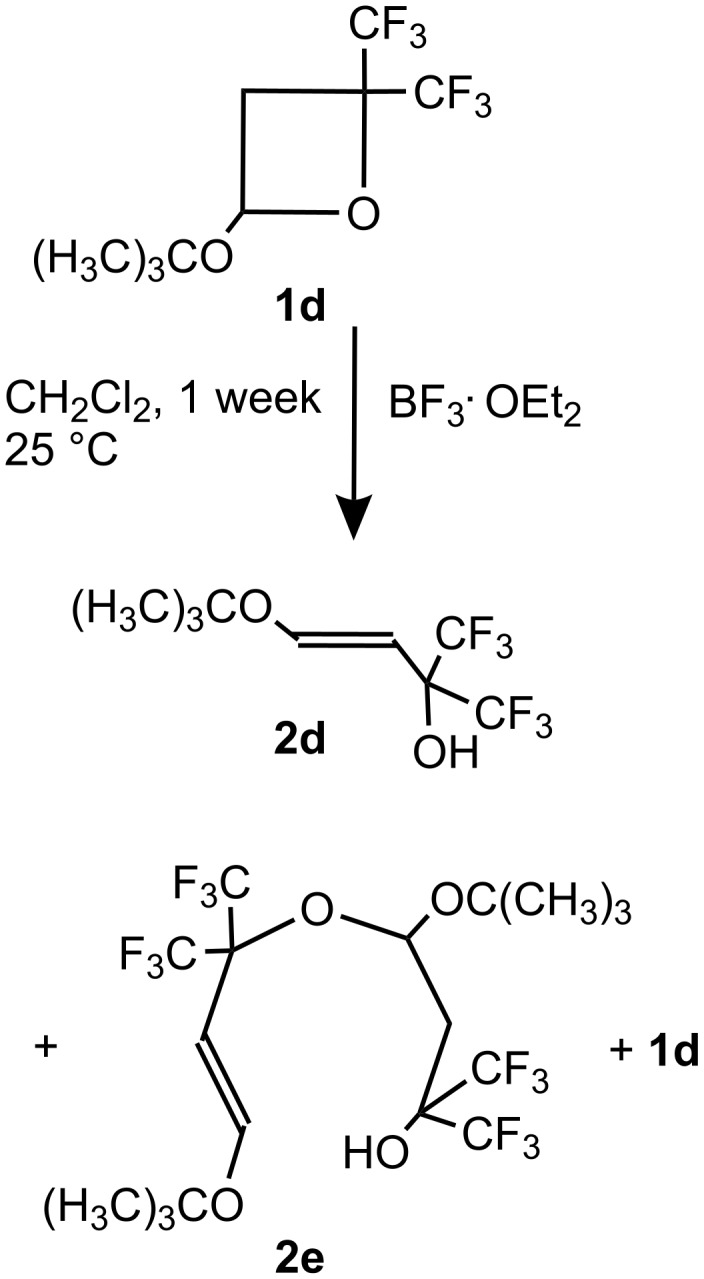
Reaction of **1d** with BF_3_·OEt_2_.

The chemical behavior of oxetane **1d** carrying the bulky *t*-C_4_H_9_O substituent is different to **1a–c**. The addition of BF_3_·OEt_2_ as catalyst to a solution of **1d** in CH_2_Cl_2_ is not exothermic and results only in a faint blue-greenish color in this case. In sharp contrast to the reaction of oxetanes **1a–c** with BF_3_·OEt_2_, this process is rather slow (85% conversion after 1 week at 25 °C) and it leads to the formation of a mixture of olefinic products **2d** and **2e** (Scheme 2).

A sample of pure **2d** was isolated by fractional distillation of the reaction mixture under reduced pressure. The structure of the olefin **2d** was established by single crystal X-ray diffraction analysis (see Supporting Information File 2).

**Scheme 3:**
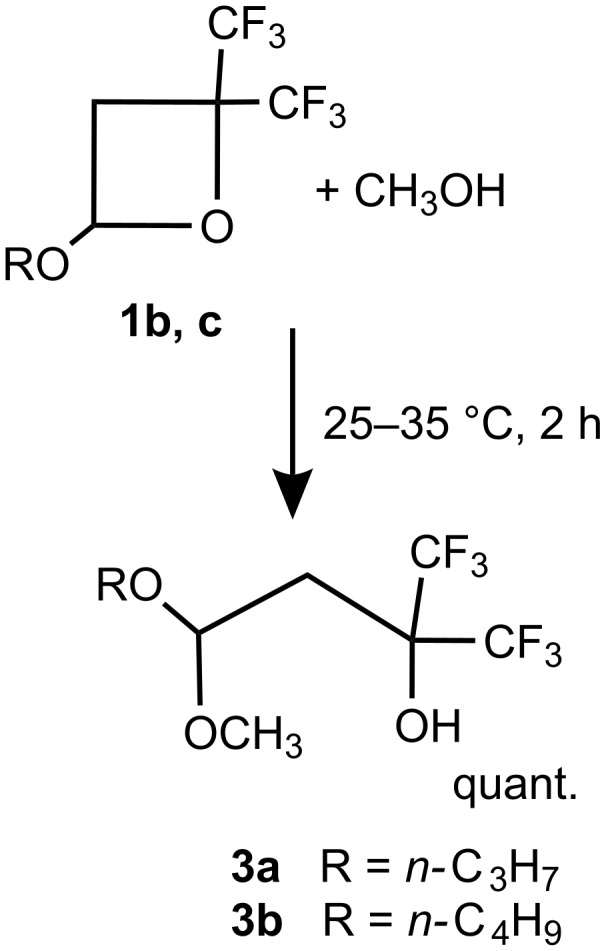
Reaction of 2,2-bis(trifluoromethyl)-4-alkoxyoxetanes **1b**, **c** with methanol.

Hydrocarbon oxetanes were reported to react with alcohols under relatively mild conditions [13]. It is interesting, that electron deficient oxetanes **1** also have similar reactivity and rapidly react with alcohols in the absence of the catalyst. The reaction leads to a ring opening with the formation of the corresponding acetals of 4,4,4-trifluoro-3-(trifluoromethyl)-3-hydroxybutanal. For example, the addition of **1b** or **1c** to an excess of methanol results in a fast and mildly exothermic reaction, leading to selective formation of acetals **3a** or **3b**, respectively (Scheme 3).

Since the vacuum distillation of **3b** lead to decomposition, the isolation of similar products in an analytically pure form was not attempted. However, removal of excess alcohol after the reaction was complete by washing with water afforded products of reasonable purity (96–98%) in >95% yield.

**Scheme 4:**
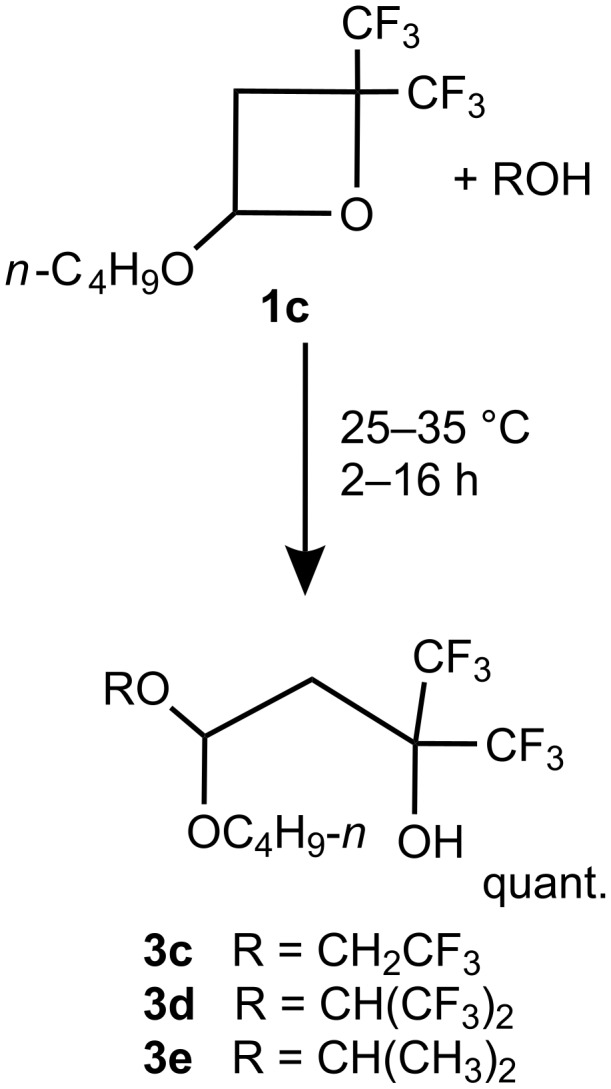
Reaction of oxetane **1c** with alcohols.

Although kinetic measurements were not carried out in this study, it appears that the reaction time and the exothermicity of the reaction of oxetanes correlates with the acidity of the corresponding alcohol. For example, in contrast to a mildly exothermic reaction of **1b**, **c** with methanol (p*K*_a_ = 15.5 [14–15]) the interaction of **1c** with more acidic CF_3_CH_2_OH (p*K*_a_ = 12.4, 12.8 [15]) or (CF_3_)_2_CHOH (p*K*_a_ = 9.3 [16]) is significantly more exothermic, leading to products **3c** and **3d**, respectively. All reactions were completed within 1–2 h at ambient temperature. On the other hand, the reaction of **1c** with the less acidic (CH_3_)_2_CHOH (p*K*_a_= 17.1 [15]) was significantly slower taking >10 h for completion at ambient temperature, as monitored by ^19^F NMR, and led to acetal **3e** (Scheme 4).

**Scheme 5:**
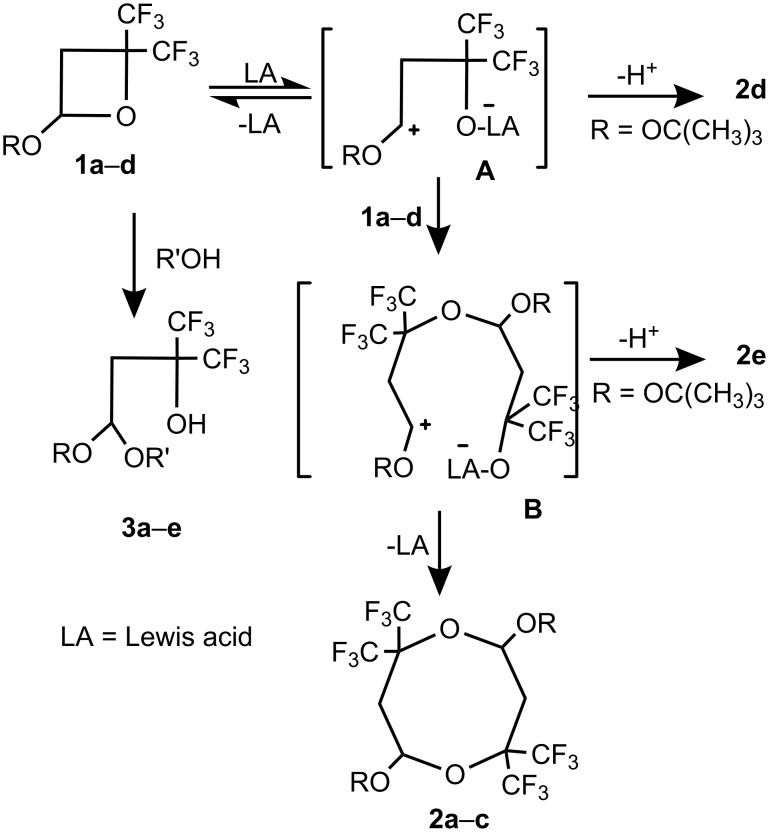
Putative mechanism for the reaction oxetanes **2a–d** with BF_3_·OEt_2_ and alcohols.

A mechanism for the reaction of these fluorinated oxetanes with Lewis acids and alcohols, is presented by Scheme 5.

Coordination of the Lewis acid with the oxetane ring oxygen results in the formation of stabilized zwitterion **A**, which probably exists in equilibrium with the starting material. The reaction of **A** with a second mole of oxetane then leads to the formation of zwitterion **B**, which can undergo intramolecular cyclization with formation of 1,5-dioxocanes **2a–c**. The Lewis acid liberated in this process is free to carry out the next catalytic cycle. It should be pointed out, that recently a zwitterion similar to **B** was observed in the isomerization of the cycloadduct of bis(trifluoromethyl)ketene and ethyl vinyl ether [9–10].

In the case of oxetane **1d**, the main channel of the reaction involves stabilization of intermediates **A** and **B** by H^+^ elimination, leading to the formation of olefins **2d** and **2e**, respectively. Such a distinct difference in the reactivity of **1d** may be a result of steric hindrance of the carbocationic center in intermediates **A** or **B**, created by the bulky (CH_3_)_3_CO-group, which favors elimination and the formation of olefins **2e** and **2d.**

The addition of alcohols to oxetanes **1a–c** probably involves the protonation of the oxetane ring oxygen atom as the first step, followed by ring opening and addition of the alkoxy anion. This mechanism agrees well with the observed order of reactivity of alcohols, with the acidic alcohols being more reactive towards the oxetane. It should be pointed out, however, that an alternative mechanism involving the “concerted” addition of the alcohol to the oxetane cannot be ruled out at this point.

**Scheme 6:**
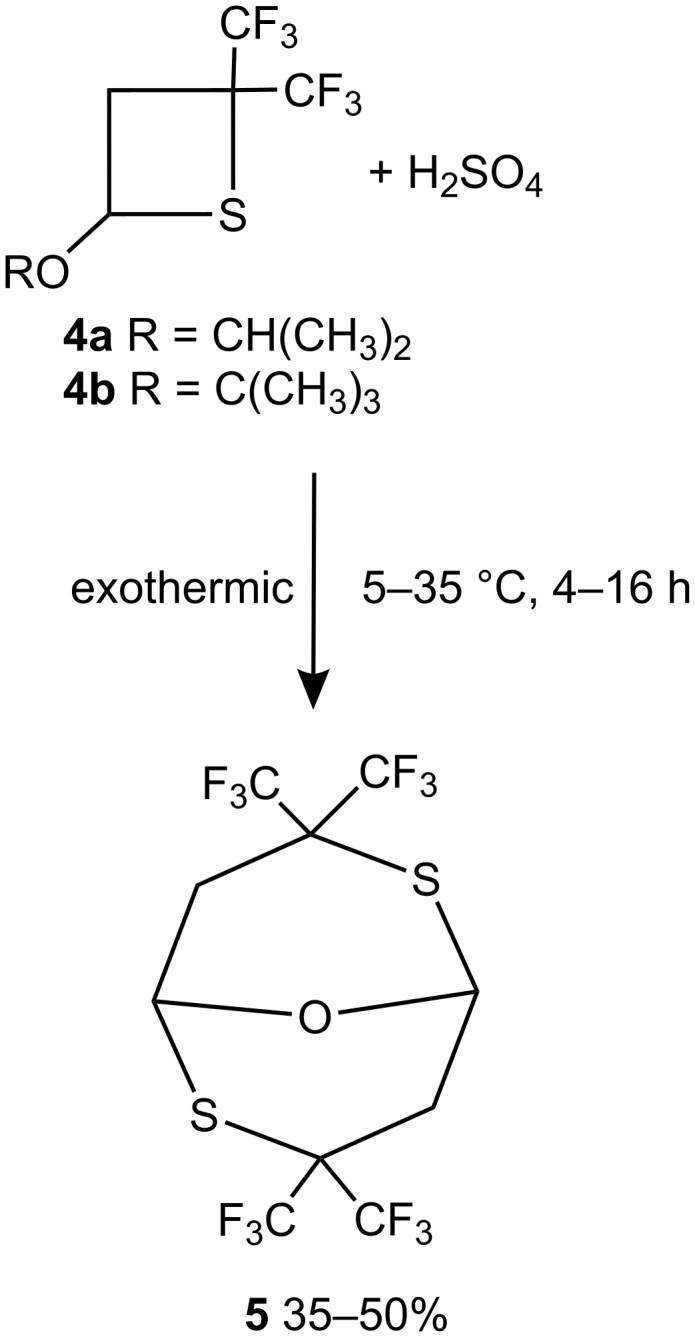
Reaction of thietanes **4a**, **b** with H_2_SO_4_ to generate **5**.

**Scheme 7:**
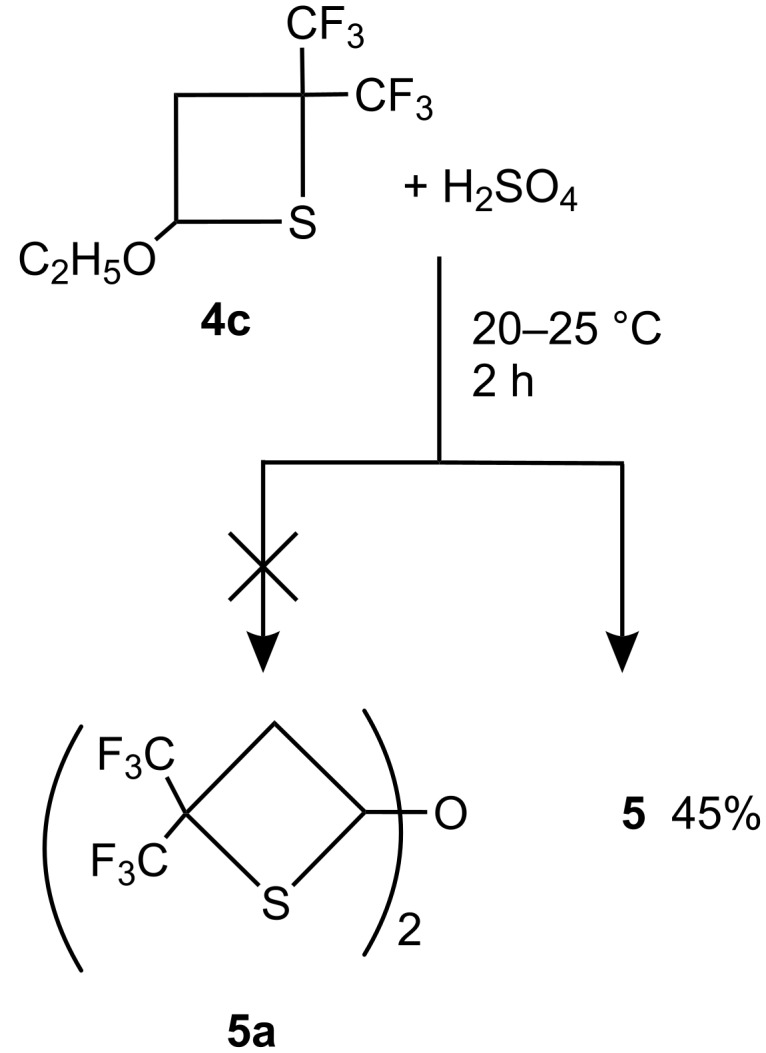
Reaction of **4c** with H_2_SO_4_.

Despite the structural similarity shared with oxetanes, thietanes **4a–c** display a totally different reactivity (Scheme 6, Scheme 7).

**Figure 2:**
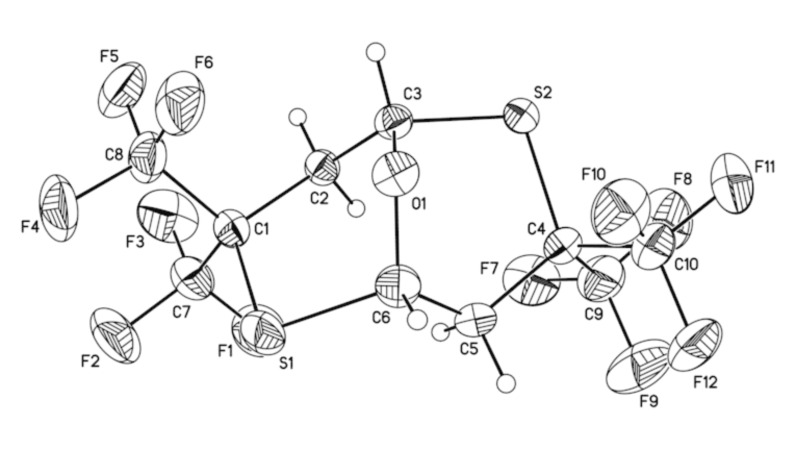
ORTEP drawing of **5** with thermal ellipsoids drawn to the 50% probability level.

For example, no reaction was detected between thietane **4a** and an excess of either methanol or hexafluoroisopropanol (25 °C, 16 h, NMR) and both **4a** and **4b** were found to be inert towards BF_3_·OEt_2_ (25 °C, 10 h, CH_2_Cl_2_, NMR). However, the addition of compounds **4a** or **4b** to concentrated H_2_SO_4_ resulted in an exothermic reaction and formation of a product, which, after single crystal X-ray diffraction analysis, was shown to be 3,3,7,7-tetrakis(trifluoromethyl)-9-oxa-2,6-dithia-bicyclo[3.3.1]nonane (**5**) (Scheme 6, Figure 2, Supporting Information File 2).

A solid product with similar melting point (91–92 °C) was observed earlier [4] in the reaction of thietanes **4** (R = CH_3_ and C_2_H_5_) with concentrated H_2_SO_4_. Based on a combination of ^1^H, ^19^F NMR, mass spectrometry and elemental analysis data, the structure of 4-[4,4-bis(trifluoromethyl)thietan-2-yloxy]-2,2-bis(trifluoromethyl)thietane **5a** was proposed for that product [4].

In order to clarify this result, thietane **4c** (R = C_2_H_5_) was treated with H_2_SO_4_ (Scheme 7) under conditions similar to those reported previously [4]. A solid product was isolated in 45% yield, which had a similar melting point and identical ^1^H, ^19^F NMR spectra to that reported for **5a** (see Supporting Information File 1, Table 1, Entry 11 and footnotes f,h), but also identical to the analytical data for compound **5** prepared from oxetanes **4a** and **4b**.

Since all experimental data obtained for the reaction of thietanes **4a–c** with H_2_SO_4_ in this work are consistent, it is concluded that the main product of this reaction is dithiocin **5**, rather than the isomeric ether **5a**, proposed in reference [4].

**Scheme 8:**
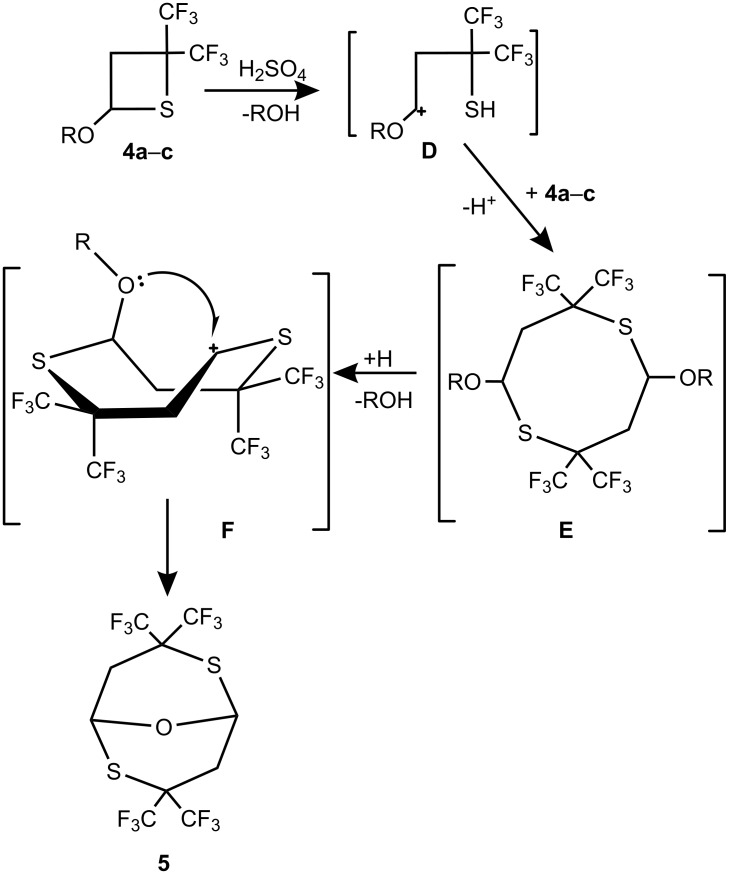
Putative mechanism for the formation bicyclic ether **5**.

A possible mechanism describing the formation of compound **5** is depicted by Scheme 8.

Protonation of the thietane sulfur of **4**, followed by ring opening, results in the formation of an oxygen stabilized carbocation **D**. Electrophilic attack of **D** at the sulfur of the second thietane molecule and loss of H^+^ would lead to the formation of intermediate **E**, structurally similar to cyloadducts **2a–c**. However, the process does not stop at this stage. Protonation of the alkoxy group of **E**, followed by the elimination of alcohol leads to the cyclic cation **F**, which further undergoes cyclization through intramolecular electrophilic attack on oxygen of the alkoxy group, resulting in the formation of 3,3,7,7-tetrakis(trifluoromethyl)-9-oxa-2,6-dithia-bicyclo[3.3.1]nonane (**5**).

## Conclusion

Despite the structural similarity, 2,2-bis(trifluoromethyl)-4-alkoxy-oxetanes and thietanes have very different reactivity towards Lewis and protic acids. While the reaction of 2,2-bis(trifluoromethyl)-4-R-oxetanes (R = C_2_H_5_, *n*-C_3_H_7_, *n*-C_4_H_9_) with BF_3_·OEt_2_ results in the fast formation of the corresponding 2,2,6,6-tetrakis(trifluoromethyl)-1,5-dioxocanes **2a–c**, the corresponding thietanes **4b**, **c** (R = *i*-C_3_H_7_, *t*-C_4_H_9_) are inert towards this Lewis acid, but rapidly react with concentrated H_2_SO_4_ with the formation of bicyclic 3,3,7,7-tetrakis(trifluoromethyl)-9-oxa-2,6-dithia-bicyclo[3.3.1]nonane **5**. Reinvestigation of a previously reported reaction of **4c**, (R = C_2_H_5_) with H_2_SO_4_, led us to the conclusion, that the product formed in this process has the structure 3,3,7,7-tetrakis(trifluoromethyl)-9-oxa-2,6-dithia-bicyclo[3.3.1]nonane (**5**), rather than 4-[4,4-bis(trifluoromethyl)thietan-2-yloxy]-2,2-bis(trifluoromethyl)-thietane (**5a**) as proposed earlier [4].

## Supporting Information

File 1 Experimental details and analytical data for compounds **2a–2e**, **3a–3e** and **5**.

File 2 X-ray data for compounds **2a**, **2b**, **2d** and **5**.
