# Supplementary material for: Acid catalyzed cyclodimerization of 2,2-bis(trifluoromethyl)-4-alkoxy-oxetanes and -thietanes. Synthesis of 2,2,6,6-tetrakis(trifluoromethyl)-4,8-dialkoxy-1,5-dioxocanes and 3,3,7,7-tetrakis(trifluoromethyl)-9-oxa-2,6-dithia-bicyclo[3.3.1]nonane
Source: Beilstein J Org Chem. 2010 May 10;6:46. doi: 10.3762/bjoc.6.46 (PMC2887279; doi:10.3762/bjoc.6.46)
Supplement: File 1 — Experimental details and analytical data for compounds 2a–2e, 3a–3e and 5. [file Beilstein_J_Org_Chem-06-46-s001.pdf]

Supporting Information

for

Acid catalyzed cyclodimerization of 2,2-bis(trifluoromethyl)-4-alkoxy- oxetanes and -thietanes. Synthesis of 2,2,6,6-tetrakis(trifluoromethyl)-4,8-dialkoxy-1,5-dioxocanes and 3,3,7,7-tetrakis(trifluoromethyl)-9-oxa-2,6-dithiabicyclo[3.3.1]nonane

Viacheslav A. Petrov*[1] and Will Marshall[2]

Address: [1]DuPont Central Research and Development (publication No 8966), Experimental Station, PO Box 800500, Wilmington DE 19880-0500, United States and [2]DuPont Corporate Center for Analytical Sciences, Experimental Station, PO Box 800500, Wilmington DE 19880-0500, United States

Email: Viacheslav A. Petrov - viacheslav.a.petrov@usa.dupont.com

*Corresponding author

Experimental details and analytical data for compounds 2a–2e, 3a–3e and 5.

Experimental Part.

^{1}H and ^{19}F NMR spectra were recorded on a Varian 500 (499.87 MHz) instrument, respectively, using $CFCl_3$ or TMS as an internal standards and $CDCl_3$ or acetone-d_6 as a lock solvent. The purity of isolated materials was established using NMR and GC and was 97–99%. GC and GC/MS analysis were carried out on a HP-6890 instrument, using HP FFAP capillary column and either TCD (GC) or mass selective (GS/MS) detectors, respectively. Hexafluoropropene, hexafluoroacetone (DuPont), CF_3CH_2OH, $(CF_3)_2CHOH$, CH_3OH, $(CH_3)_2CHOH$, $BF_3{\cdot}Et_2O$, H_2SO_4 (96%) (Aldrich) and were used without further purification.

Oxetanes **1a–d** were prepared using a slightly modified literature procedure [1] by bubbling hexafluoracetone into a solution of the vinyl ether in CH_2Cl_2 at 10–25 °C, followed by the removal of solvent under reduced pressure. The crude products (purity >97%, NMR) were stored refrigerated, over a small amount of solid K_2CO_3 to prevent polymerization and used for further reactions without further purification. Thietanes **4a–c** were prepared according to literature procedures [2].

Crystallography:

X-ray data for **2a, 2b, 2d** and **5** were collected at −100 °C using a Bruker 1K CCD system equipped with a sealed tube molybdenum source and a graphite monochromator. The structures were solved and refined using the SHELXTL [3] software package, refinement by full-matrix least squares on F^2, scattering factors from Int. Tab. Vol C Tables 4.2.6.8 and 6.1.1.4. Crystallographic data (excluding structure factors) for the structures in this paper have been deposited with the Cambridge Crystallographic Data Centre as supplementary publication nos. CCDC #761670 to #761573. Copies of the data can be obtained, free of charge, on application to CCDC, 12 Union Road, Cambridge CB2 1EZ, UK, (fax: +44 1223 336033 or e-mail: deposit@ccdc.cam.ac.uk).

Reaction of oxetanes 1a–c with BF_3·Et_2O.

To a stirred solution of 0.5–3 g of oxetane **1a, 1b** or **1c** in 10–15 ml of CH_2Cl_2 was added 1–3 drops of BF_3·Et_2O at ambient temperature. After the addition of the catalyst, the appearance of an intense dark blue color was observed and the reaction temperature rose to 30–35 °C. Over the next 15–30 min the color of the reaction mixture went from blue to dark red and finally brown. At this point the formation of a brown precipitate was observed. The reaction mixture was cooled to −70 °C, filtered cold and the filter cake washed several times with water and then with a small amount of cold hexane to give 0.2–1.5 g of white or slightly yellow solids, compounds **2a–c**. Analytically pure samples of **2a–c** were prepared by crystallization from hexane. Melting points and NMR and elemental analysis data for **2a–c** are given in Table 1.

Reaction of oxetane 1d with BF_3·Et_2O.

To a stirred solution of 26.6 g (0.1 mol) of oxetane **1d** in 50 ml of CH_2Cl_2 was added 3 drops of BF_3·Et_2O at ambient temperature. Addition of the catalyst was not exothermic. The reaction mixture was stirred for 7 days at ambient temperature. At this point the conversion of **1d** was ~85% (NMR, ratio of **1d**:**2d**:**2e** – 15:15:70). The reaction mixture was washed with water (2 x 100 ml), organic layer separated, dried over $MgSO_4$ and the solvent was removed under vacuum (~100 mm Hg, <20 °C). The residue was distilled at 20 mmHg to give two fractions:

- 5 g of material bp 30–40 °C/5mmHg (olefin **2d,** *trans*-isomer, purity >98%, isolated yield 19%), crystallized on standing, mp 69–70 °C;
- 12 g of material bp 50–55 °C/5mmHg – mixture of liquid and solid materials, based on NMR mixture of **1d**, **2e** and **2d** in ratio 16:16:68. Calculated yield of **2d** - 31%).
- In the cold trap 4 g of **1d** (purity 90%, contaminated by an equal amoumt of **2d** and **2e**) was collected. Spectroscopic data for **2e** and **2d** are given in Table 1.

Reaction of oxetanes 1b, **c with alcohols.**

Oxetane **1** (0.02 mol) was added slowly to 20 ml of the corresponding alcohol at such a rate that the internal temperature was kept at <30 °C. The reaction mixture was stirred for

2–16 h at ambient temperature and then diluted with 200 ml of water. The organic layer was separated, washed with water, dried over $MgSO_4$ and analyzed. NMR data for compounds **3a–e** are given in Table 1.

Reaction of thietanes 4a, **b with H_2SO_4.**

Thietane **4a** (5.4 g, 0.02 mol) or **4b** (5.6 g, 0.02 mol) was added to 25 ml of of 96% H_2SO_4 at such a rate that the reaction temperature was kept between 25–35 °C. The reaction mixture was stirred at ambient temperature for 2–16 h, poured onto crushed ice and extracted with 50 ml of CH_2Cl_2. The organic layer was washed with 10% solution of $NaHCO_3$, dried over $MgSO_4$, the solvent was removed under vacuum and the solid residue recrystallized from hexane to give 1.65 g (35%) and 2.4 g (50%) of **5**, mp 90–91 °C (from **4a**) and 91–92 °C (from **4b**), respectively. Data of ^{1}H, ^{13}C and ^{19}F NMR spectroscopy are given in Table 1. GC/MS (*m/z*, in CH_2Cl_2, relative intensity %): 434 (M^+, $C_{10}H_6F_{12}OS_2^+$, 35%), 270 ($C_6H_4F_6OS_2^+$, 30%), 241 (40%), 209 ($C_5H_3F_6S^+$, 100%), 189, 171, 145 ($C_4H_2F_5^+$), 113, 69(CF_3^+).

Reaction of thietane 4c with H_2SO_4

6.3 g (0.025 mol) of **4c** was added in small portions to 25 ml of 96% H_2SO_4 at such a rate that the temperature of the reaction mixture was kept at <20 °C. The reaction mixture was stirred at ambient temperature for 1 h, poured onto crushed ice and extracted with 50 ml of CH_2Cl_2. The organic layer was washed with 10% solution of $NaHCO_3$, dried over $MgSO_4$, the solvent was removed under vacuum and the solid residue recrystallized from hexane to give 2.8 g (45%) of **5** mp 91–92 °C. ^{1}H, ^{13}C and ^{19}F NMR spectra of the material obtained were identical to the spectra of samples prepared from **4a** and **4b**.

Table 1: Yields, boiling (melting) points and NMR data for compounds **2a–e**, **3a–e** and **5**.

**Entry No**	**Comp.**	**Yield (%)**	**bp (°C/mmHg) (mp °C)**	**^{19}F NMR[a] (δ, ppm, *J*, Hz)**	**^{1}H NMR[a] (δ, ppm, *J*, Hz)**
1	**2a**[b,c]	31	(127–128)	−76.06 (3F, q, 10.5)<br>−77.15 (3F, q, 10.5)	1.2 (3H, t, 7.2)<br>2.61 (1H, d, 17.1)<br>3.04 (1H, dd, 17.1, 5.9)<br>3.63 (1H, m)<br>3.83 (1H, m)<br>5.65 (1H, d, 5.8)
2	**2b**[b,d]	34	(68–69)	−75.63 (3F, q, 10.2)<br>−76.60 (3F, q, 10.2)	0.95 (3H, t, 7.6)<br>1.60 (2H, m)<br>2.58 (1H, d, 16.8)<br>2.84 (1H, dd, 16.8, 5.5)<br>3.40 (1H, m)<br>3.72 (1H, m)<br>5.53 (1H, d, 5.5)
3	**2c**[b,e]	42	(54–55)	−75.63 (3F, q, 10.2)<br>−76.60 (3F, q, 10.2)	0.95 (3H, t, 7.2)<br>1.41 (2H, m)<br>1.59 (2H, m)<br>2.57 (1H, d, 17.3)<br>2.84 (1H, dd, 17.3, 6.2)<br>3.45 (1H, m)<br>3.77 (1H, m)<br>5.53 (1H, d, 5.6)

4	**2d**	30	(69–70)	−78.98 (s)	1.35 (9H, s)<br>2.90 (1H, s)<br>5.06 (1H, d, 12.1)<br>7.00 (1H, d, 12.1)
5	**2e**	—	43–55/10	−73.14 (3F, q, 11.2)<br>−73.83 (3F, q, 11.2)<br>−75.82 (3F, q, 10.0)<br>−79.79 (3F, q, 10.0)	1.37 (9H, s)<br>2.24 (1H, dm, 15.7)<br>2.46 (1H, dd, 15.7, 2.8)<br>5.03 (1H, d, 12.6)<br>6.61 (1H, d, 9.1)<br>5.71 (1H, s)<br>7.03 (1H, d, 12.6)
6	**3a**	—	—	−77.82 (3F, q, 10.2)<br>−78.16 (3F, q, 10.2)	0.95 (3H, t, 7.5)<br>1.66 (2H, m)<br>2.24 (2H, d)<br>3.50 (3H, s)<br>3.53 (1H, m)<br>3.70 (1H, m)<br>4.85 (1H, t, 5.3)<br>5.70 (1H,s)

7	**3b**	—	—	−77.31 (3F, q, 10.2)<br>−77.72 (3F, q, 10.2)	0.94 (3H, t, 7.5)<br>1.40 (2H, m)<br>1.60 (2H, m)<br>2.23 (2H, d, 6)<br>3.43 (3H,s)<br>3.53 (1H, m)<br>3.73 (1H, m)<br>4.83 (1H, t, 5.3)<br>5.68 (1H, s)
8	**3c**	—	—	−74.82 (3F,t, 7.9)<br>−76.89 (3F, q, 10.2)<br>−78.87 (3F, q, 10.2)	0.97 (3H, t, 7.4)<br>1.41 (2H, m)<br>1.65 (2H, m)<br>2.31 (2H, m)<br>2.84 (1H, dd, 17.3, 6.2)<br>3.58 (1H, m)<br>3.84 (1H, m)<br>5.08 (1H, dd, 5.4, 7.2)<br>5.26 (1H, s)
9	**3d**	—	—	−73.28 (3F,dq, 9.5, 5.7)<br>−77.57 (3F,dq, 9.5, 5.7)<br>−76.20 (3F, q, 9.5)<br>−78.72 (3F, q, 9.5)	0.95 (3H, t, 7.5)<br>1.39 (2H, m)<br>1.64 (2H, m)<br>2.35 (2H, m)<br>3.58 (1H, m)<br>3.92 (1H, m)<br>4.39 (1H, sept, 5.5)

					5.00 (1H, s) 5.13(1H, dd, 8.0, 4.5)
10	**3e**	—	—	−77.40 (3F, q, 10.0) −77.54 (3F, q, 10.0)	0.92 (3H, t, 7.0) 1.18 (3, d, 6.0) 1.22 (3, d, 6.0) 1.38 (2H, m) 1.57 (2H, m) 2.20 (2H, d, 6.0) 3.47 (1H, m) 3.67 (1H, m) 3.93 (1H, sept, 6.0) 4.93 (1H, t, 6.0) 5.89 (1H, s)
11	**5**	35–50	(91–92)[f]	−71.25 (3F,q, 11.0) −72.31 (3F,q, 11.0)[g,h]	2.83 (1H, ddq, 16.6, 8.4, 1.6) 3.42 (1H, dd, 16.6, 8.9) 5.96 (1H, t, 8.5)[g,i]

[a] in $CDCl_3$

[b] Elemental analysis: Found (calcd) % for:

2a: C = 35.08 (35.31), H = 3.10 (3.39), F = 47.64 (47.87)

2b: C = 38.02 (38.11), H = 3.80 (4.00), F = 44.99 (45.21)

2c: C = 40.59 (40.61), H = 4.62 (4.54), F = 42.60 (42.82)

[c] ^{13}C NMR ($CDCl_3$): 14.74, 36.94, 63.69, 79.06 (sept, J = 28.8 Hz), 96.90, 122.34 (q, J = 290 Hz), 122.72 (q, J = 290 Hz) ppm.

[d] ^{13}C {H}NMR ($CDCl_3$): 13.54, 19.00, 31.10, 67.94, 79.15 (sept, *J* = 28.3 Hz), 96.90, 122.34(q, *J* = 290 Hz), 122.68 (q, J=290 Hz) ppm.

[e] ^{13}C{H} NMR ($CDCl_3$): 10.21, 22.44, 37.00, 69.19, 69.86, 79.13 (sept, *J* = 28.3 Hz), 97.10, 122.34 (q, *J* = 290 Hz), 122.71 (q, *J* = 290 Hz) ppm.

[f] reported for compound **5a** : mp 91–92 °C [2].

[g] in acetone-d_6

[h] NMR data reported for compound **5a**: ^{1}H NMR ($CDCl_3$) : 2.72 (1H, dd, *J* = 15.0 Hz), 3.05 (1H, dd, *J* = 15.0, 5.0 Hz), 5.45(1H, t, *J* = 9.0 Hz) ppm; ^{19}F (CCl_4): −70.0 (m) ppm [2].

[i] ^{13}C{H} NMR: (acetone-d_6): 29.47, 55.18 (sept, *J* = 28.3 Hz), 69.77, 123.50 (qq, *J* = 282, 1.5 Hz), 123.85 (q, *J* = 282 Hz) ppm.

References

[1] Gambaryan, N. P.; Simonyan, L. A.; Petrovskii, P. V. *Izv. Akad. Nauk SSSR, Ser. Khim.* **1967,** 918–921.

[2] Kitazume, T.; Otaka, T.; Takei, R.; Ishikawa, N. *Bull. Chem. Soc. Jpn.* **1976,** *49,* 2491–2494.

[3] Sheldrick, G.; SHELXTL Sofware Suite, v. 6.0, Bruker Axes Corp.: Madison WI, 1996.
